# Train Hunting Related Fast Degradation of a Railway Crossing—Condition Monitoring and Numerical Verification

**DOI:** 10.3390/s20082278

**Published:** 2020-04-17

**Authors:** Xiangming Liu, Valéri L. Markine

**Affiliations:** Department of Engineering Structures, Delft University of Technology, 2628 CN Delft, The Netherlands

**Keywords:** railway crossing, wheel-rail impact, train hunting, numerical verification, railway track maintenance

## Abstract

This paper presents the investigation of the root causes of the fast degradation of a railway crossing. The dynamic performance of the crossing was assessed using the sensor-based crossing instrumentation, and the measurement results were verified using the multi-body system (MBS) vehicle-crossing model. Together with the field inspections, the measurement and simulation results indicate that the fast crossing degradation was caused by the high wheel-rail impact forces related to the hunting motion of the passing trains. Additionally, it was shown that the train hunting was activated by the track geometry misalignment in front of the crossing. The obtained results have not only explained the extreme values in the measured responses, but also shown that crossing degradation is not always caused by the problems in the crossing itself, but can also be caused by problems in the adjacent track structures. The findings of this study were implemented in the condition monitoring system for railway crossings, using which timely and correctly aimed maintenance actions can be performed.

## 1. Introduction

**Figure 1:**
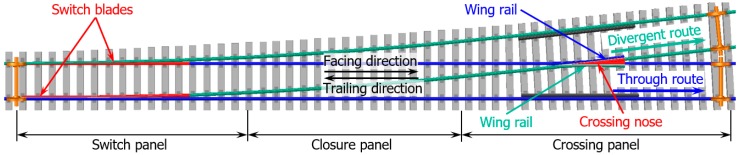
Standard left-hand railway turnout and the definition of the passing routes.

In the railway track system, turnouts (switches and crossings) are essential components that allow trains to pass from one track to another. A standard railway turnout is composed of three main parts: switch panel, closure panel, and crossing panel, as shown in Figure 1. In a railway turnout, the crossing panel is featured to provide the flexibility for trains to pass in different routes.

**Figure 2:**
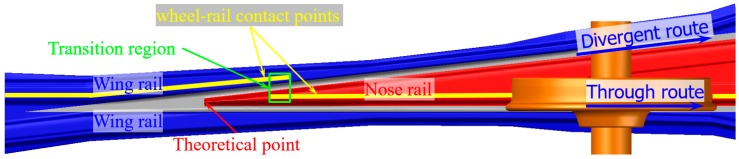
Wheel-rail interaction in the railway crossing for through route traffic.

For rigid crossings that are commonly used in conventional railway lines, the gap between the wing rail and the nose rail usually results in high wheel-rail impacts in the transition region where the wheel load transits from the wing rail to the nose rail (vice versa, Figure 2), which makes the crossing a vulnerable spot in the railway track. In the case of crossings that are mainly used for the through route traffic (e.g., crossings in the crossover), there is no specific speed limit [1] and trains can pass through the crossings with a high velocity of up to 140 km/h. The high train velocity makes the wheel-rail impact more serious. In the Dutch railway system, around 100 crossings are urgently replaced every year [2] due to unexpected fatal defects, which not only result in substantial maintenance efforts, but also lead to traffic disruption and can even affect traffic safety.

In contrast to a switch panel, wherein sensors are instrumented for condition monitoring [3,4] and remaining useful life prediction [5], monitoring in a crossing panel is usually absent. As a result, the real-time information on the condition of railway crossings is limited. The present maintenance activities are mainly reactive and based on the experience of the contractors. In this case, the root causes of the crossing degradation are not always resolved by the maintenance actions, and the crossings are likely to be operated in a degraded condition. To improve this situation, necessary guidance for maintenance actions is highly required.

Proper crossing maintenance usually relies on condition assessment and degradation detection, which can be realized through field monitoring. In recent years, condition monitoring techniques have been frequently applied in the railway industry. Aside from the above-mentioned instrumentation on the turnout switches, vehicle-based monitoring systems have been applied in track stiffness measurement [6] and estimation [7], track alignment estimation [8], hanging sleepers detection [9], and track fault detection [10], etc. Compared with the normal track, the current studies on railway crossings are mainly based on numerical simulation. Typical contributions include wheel-rail interaction analysis [11,12,13,14,15,16,17,18,19,20,21], damage analysis [16,17,22,23], and prediction [18,24,25] as well as crossing geometry and track stiffness optimization for better dynamic performance [16,26]. Field measurements are mainly used for the validation of numerical models. The monitoring of railway crossings for condition assessment and degraded component detection is still limited.

In the previous study, key indicators for the crossing condition assessment based on the field measurement were proposed [27,28]. Additionally, a numerical vehicle-crossing model was developed using a multi-body system (MBS) method to provide the fundamental basis for the condition indicators [29]. In this study, the condition indicators, as well as the MBS model, were applied in the condition monitoring of a fast degraded railway crossing. The main goals of this study were to investigate the root causes of the crossing degradation as well as to assess the effectiveness of the current maintenance actions.

Based on the objectives, this paper is presented in the following order. The experimental and numerical tools, including the crossing condition indicators, are briefly introduced in Section 2. The measurement results and the crossing degradation analysis as well as the effectiveness of the current maintenance actions are presented in Section 3 and Section 4. Based on the measurement results and field inspections, the root causes for the fast crossing degradation were investigated with the assistance of the MBS model, as presented in Section 5. In Section 6, the verification of the effectiveness of the maintenance actions is given. Finally, in Section 7, major conclusions are provided.

## 2. Methodology

In this section, the experimental tools for the crossing condition monitoring, as well as the indicators for the crossing condition assessment, are briefly introduced. The MBS vehicle-crossing model for the verification of the experimental findings is also presented.

### 2.1. Experimental Tools

The experimental tools mainly consisted of the in-site instrumentation system modified from ESAH-M (Elektronische System Analyse Herzstückbereich-Mobil) and the video gauge system (VGS) for wayside monitoring, as briefly described below. Both tools have already been introduced and actively applied in previous studies. Detailed information regarding the installation and data processing can be found in [27,30].

#### 2.1.1. Crossing Instrumentation

**Figure 3:**
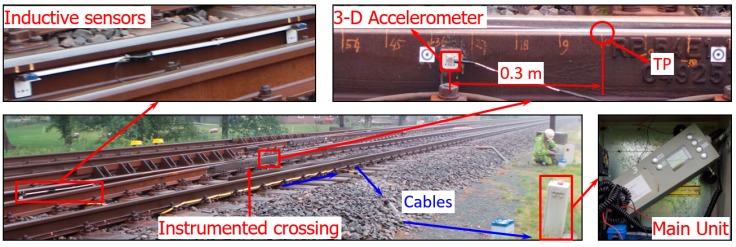
Crossing instrumentation based on ESAH-M.

The main components of the crossing instrumentation are an accelerometer attached to the crossing nose rail for 3-D acceleration measurement, a pair of inductive sensors attached in the closure panel for train detection as well as train velocity calculation, and the main unit for data collection. An overview of the instrumented crossing is shown in Figure 3.

The main outputs of the crossing instrumentation were the dynamic responses of the crossing nose, including the wheel-rail impact accelerations and locations, etc. All these responses were calculated within the transition region, which can be obtained through field inspection [29]. Based on these measured responses and the correlation analysis between the responses [28], two critical condition indicators related to the wheel impact and fatigue area, respectively, were proposed.

**Figure 4:**
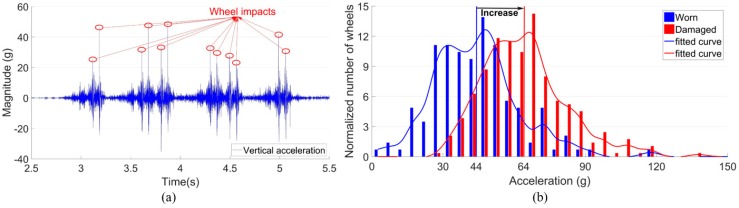
Indicator for the wheel impact. (**a**) Procedure for the obtainment of wheel impacts. (**b**) Example of the variation of the wheel impacts in different condition stages.

The wheel impact is reflected by the vertical accelerations, which were obtained from the crossing and processed through statistical analysis. This indicator is mainly based on the magnitude of the impacts due to each passing wheel (Figure 4a), and the changes in time indicate the different condition stages of the crossing (Figure 4b).

**Figure 5:**
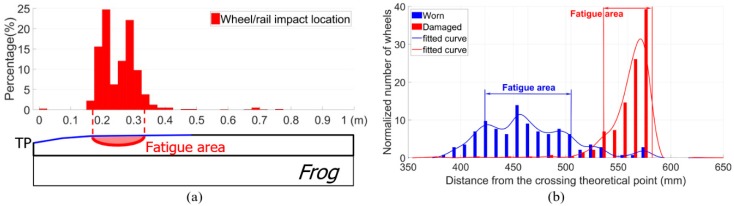
Demonstration of the crossing fatigue area detection. (**a**) Definition of the fatigue area. (**b**) Example of the fatigue area changes in different crossing condition stages.

The fatigue area is defined as the region where the majority of wheel impacts are located on the crossing, and where ultimately the crack initiates (Figure 5a). In practice, the fatigue area can be simplified as the confidence interval of [*a* − *σ*, *a* + *σ*], where *a* is the mean value of the wheel-rail impact locations, and *σ* is the standard deviation. The location and size of the fatigue area are critical values for the assessment of crossing wear and plastic deformation. A wide fatigue area usually represents well-maintained rail geometry. As demonstrated in Figure 5b, when the crossing condition was degraded from “Worn” to “Damaged”, the fatigue area was dramatically narrowed and shifted further from the theoretical point (TP) of the crossing. More information about the fatigue area can be found in the previous study [27].

#### 2.1.2. Wayside Monitoring System

**Figure 6:**
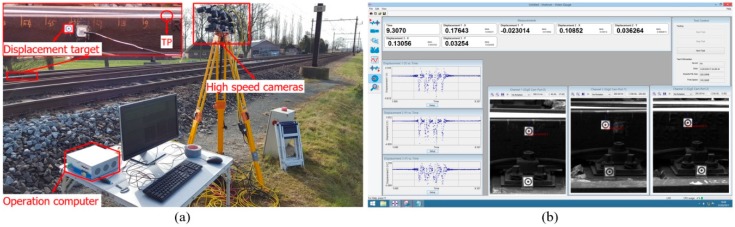
Wayside monitoring. (**a**) System setup. (**b**) The screen of displacement measurements.

The VGS for wayside monitoring is a remote measurement device based on digital image correlation (DIC). It uses high-speed digital cameras to measure the dynamic movements of the selected targets in the track. The system, set up together with the targets installed on the crossing rail next to the instrumented accelerometer, is shown in Figure 6a, and the demo of the displacement measurement is shown in Figure 6b. The main outputs are the vertical displacements of the tracked targets with a stable sampling frequency of up to 200 Hz.

**Figure 7:**
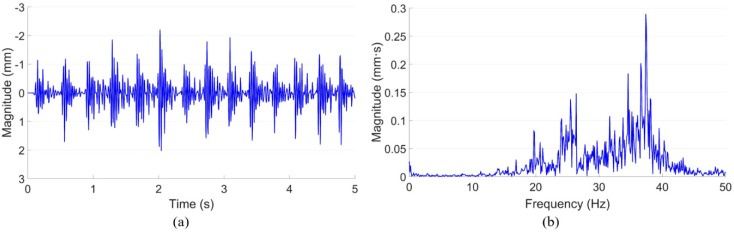
Ground activated camera vibration. (**a**) Time domain signal. (**b**) Frequency domain responses.

Due to the limitation of the experimental conditions, the wayside monitoring system is usually set up close by the side of the track, which will introduce extra noise in the measured displacement results. To improve the accuracy of the measurement, the noise part needs to be eliminated. The noise mainly comes from the ground-activated camera vibration, which can be manually activated by hammering the ground near the camera. The measured camera vibrations in both the time and frequency domains are given in Figure 7.

**Figure 8:**
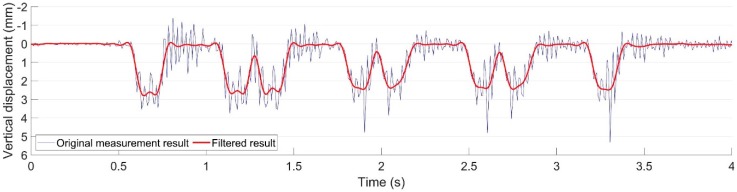
Examples of the measured rail vertical displacement.

Despite the differences in the displacement responses in the two monitored crossings, the main resonance of the camera vibration was around 15–45 Hz. In the previous study [30], the main components in the displacement signal were elaborated. The train-track components related to displacement responses are mainly distributed below 10 Hz, which do not overlap with the camera vibration introduced noise. The noise part due to camera vibration can then be reduced through low-pass filtering, as shown in Figure 8.

The magnitude of the dynamic vertical displacement of the rail directly reflects the intensity of the track movement due to the passing trains. By comparing the measured rail displacement with the reference level, which can be obtained from numerical simulation using the parameters in the designed condition, the ballast settlement level of the monitored location can be estimated. The MBS model for the crossing performance analysis is described later in this section.

### 2.2. Multi-Body System (MBS) Vehicle-Crossing Model

**Figure 9:**
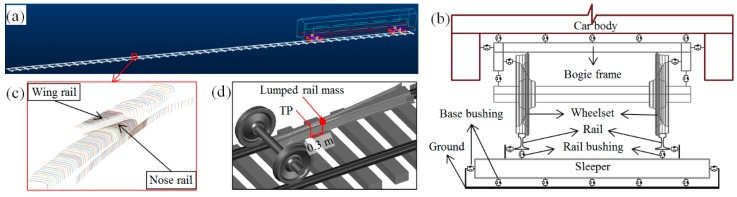
Multi-body system (MBS) model. (**a**) Vehicle-track model. (**b**) Flexible connections in the model. (**c**) Crossing profiles. (**d**) Rail element for acceleration extraction.

The numerical model for the crossing performance analysis was developed using the MBS method VI-Rail (Figure 9a). The rail pads, clips, and ballast were simulated as spring and damping elements (rail busing and base busing, Figure 9b). In the vehicle model, the car body, bogie frames and the wheelsets were modeled as rigid bodies with both the primary suspension and secondary suspension taken into account (Figure 9b). The track model was a straight line with the crossing panel (Figure 9c) situated in the middle of the track. The rail element for the acceleration and displacement extraction was the lumped rail mass located 0.3 m from the TP of the crossing (Figure 9d), which is consistent with the setup of the field measurements (Figure 3 and Figure 6a).

The detailed model development, experimental validation, and numerical verification can be found in the previous study [29]. Corresponding to the condition indicators, the main outputs of the MBS model are the wheel impact acceleration, transition region and wheel-rail contact forces. Using the MBS model, the condition of the monitored crossing, as well as the detected track degradations, can be verified.

## 3. Field Measurements and Analysis

**Figure 10:**
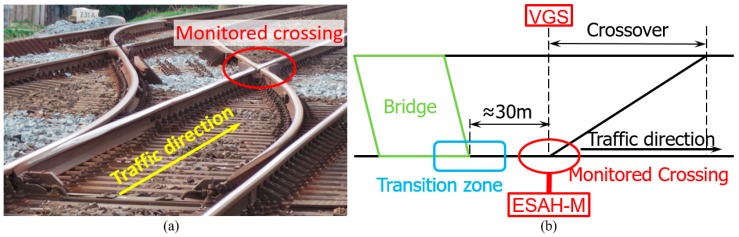
Overview of the monitored crossing. (**a**) On-site view. (**b**) Sketch view.

The monitored crossing was a cast manganese crossing with an angle of 1:9. As part of a crossover, trains mainly pass the crossing in the facing through route (Figure 2) with a velocity of around 140 km/h. The on-site view of the crossing is shown in Figure 10a. According to the maintenance record, this crossing was suffering from fast degradation with the service life of only around three years (18 years on average [2]). At the beginning of the condition monitoring, the damaged crossing was completely renovated. 

Figure 10b gives a sketch view of the crossing, including the setup of the monitoring devices and the layout of the adjacent structures, especially the small bridge in front of the crossing. Considering that the bridge is located quite close to the monitored crossing, the performance of the crossing might be affected by the bridge, which will be discussed later.

The measurement results from the crossing instrumentation were based on multiple train passages in one monitoring day. For the wayside monitoring, one sufficient train passage is enough to estimate the ballast condition. To maximally reduce the influence of the vehicle-related variables, the selected results were restricted to the commonly operated VIRM trains with velocities of around 140 km/h.

### 3.1. Wheel Impacts

**Figure 11:**
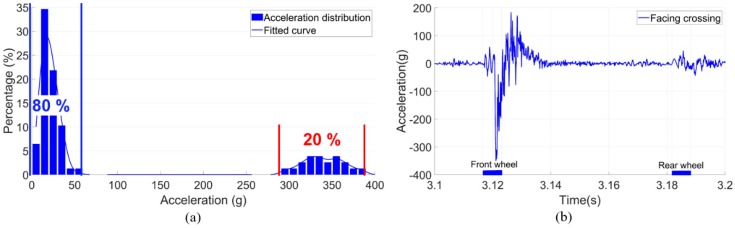
Vertical acceleration responses of the monitored crossings. (**a**) Distribution based on multiple train passages in one day. (**b**) Example of impacts due to one bogie.

Based on the estimated transition regions, the wheel impact accelerations were calculated. The distribution of the wheel impacts due to multiple wheel passages is shown in Figure 11a. The sample size, in this case, was 78 passing wheels. It can be seen that the wheel impacts presented a bimodal distribution. Around 80% of the wheel impacts were below 50 g, while the remaining 20% of the wheel impacts were extremely high with a mean value of around 350 g. Such a polarized distribution of impacts indicates the highly unstable wheel-rail interaction in this crossing. It was demonstrated in a previous study [29] that for this type of railway crossing, the average level of the wheel impact is around 50 g, meaning that the 20% of high impacts of the monitored crossing are already more than seven times higher than the average impact level. It can be imagined that such high impacts will dramatically accelerate the degradation procedure of the crossing.

An example of the impact acceleration response in the time-domain due to the first bogie of a VIRM train is shown in Figure 11b. It can be seen that for the two passing wheels from the same bogie, the impacts can be quite different. The impact due to the front wheel was up to 350 g, while the rear wheel activated vertical acceleration was only 20 g. It has to be noted that the high impacts were not always introduced by the front wheel, but appeared to have random occurrences. Such results further confirmed the instability of wheel-rail interaction at this crossing.

### 3.2. Fatigue Area

**Figure 12:**
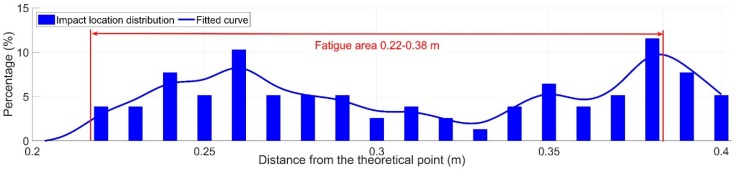
Measured fatigue area of the monitored crossing.

The measured fatigue area of the monitored crossing is presented in Figure 12. It can be seen that the wheel impacts were widely distributed at 0.22–0.38 m from the TP with the fatigue area size of 0.16 m. According to the previous study [28], the transition region (Figure 2) for this type of crossing is around 0.15–0.4 m. The fatigue area widely covered 64% of the transition region, which can be considered to be in line with the expectation of a new crossing profile. Such results further confirmed that the crossing rail was not worn or deformed.

It has to be noted that the fatigue area does not conform to the normal distribution (referring to the “Worn” stage demonstrated in Figure 5b). Combined with the results of the wheel impacts such a fatigue area further confirmed the instability of the wheel-rail contact in the monitored crossing.

In a previous study [27], it was found that the crossing degradation was accompanied by the increase of wheel-rail impacts and the reduction in the fatigue area. The large number of extremely high wheel-rail impacts and relatively wide fatigue area clearly indicate the abnormal performance of the monitored crossing. Finding the root causes of such abnormality is the key to improving the dynamic performance of the crossing.

### 3.3. Ballast Settlement

**Figure 13:**
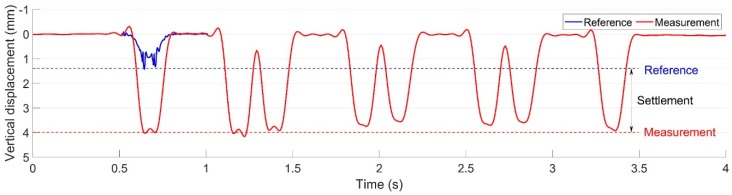
Ballast settlement in the monitored crossing.

The measured vertical displacement of the crossing rail is presented in Figure 13. It can be seen that the vertical rail displacement was around 4 mm. The measured displacement result can be considered to have two main parts: the elastic deformation and the gap between the sleeper and ballast. Considering that the ballast settlement is the accumulated effect due to multiple wheel passages, the plastic deformation caused by each passing train can be neglected. Due to the high impacts in the crossing panel, the ballast is usually settled unevenly, which results in hanging sleepers. Using the validated MBS model, it was calculated that the rail displacement in the reference condition was 1.4 mm (Figure 13), which only consisted of the elastic deformation part. By comparing these two results, it could be calculated that the gap between the sleeper and ballast was 2.6 mm, which can be estimated as the settlement of ballast. It was observed that the rail displacement obtained from the MBS simulation was much higher than that in a normal track (less than 1 mm [27,31]), which indicates the vulnerability of the ballast in the railway crossings.

In a previous study [27], it was found that track irregularities such as rail joints and turnout crossings can lead to the fast deterioration of the ballast, and the ballast settlement will in turn accelerate the degradation procedure of other related track components. In this study, the 2.6 mm ballast settlement was already higher than those in the previously monitored welded joints (≈1.5 mm) and movable crossings (≈2 mm), which revealed the seriously deteriorated ballast condition.

It can be concluded that the monitored crossing was suffering from rapidly occurring, extremely high wheel-rail impacts and severe ballast settlement. For a recently renovated crossing, such performance is quite abnormal.

## 4. Effectiveness Analysis of the Maintenance Actions

The constantly occurring extremely high wheel-rail impacts as well as serious ballast settlement clearly indicate the degraded condition of the crossing. In order to improve such a situation, various maintenance actions were implemented in this location including ballast tamping, fastening system renovation, etc. In this section, the effectiveness of the maintenance actions are briefly discussed, as presented below.

### 4.1. Ballast Tamping

**Figure 14:**
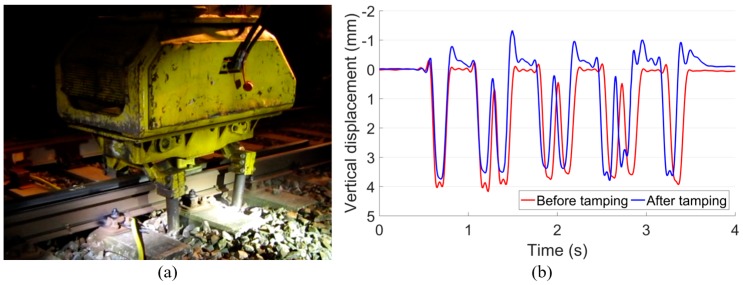
(**a**) Squeezing machine used for ballast tamping in the monitored crossing. (**b**) Measured rail displacement before and after ballast tamping.

Considering that the crossing rail was lately renovated with limited wear or plastic deformation, the severe ballast settlement was suspected to be the main cause for the high wheel-rail impacts. Therefore, ballast tamping actions were frequently performed in this location by the local contractor. However, due to the lack of maintenance facilities, the tamping actions were mainly performed using the squeezing machine (Figure 14a) without track geometry correction. It can be imagined that the settled ballast cannot be fully recovered with such tamping action. As shown in Figure 14b, after tamping, the rail displacement was not dramatically reduced.

**Figure 15:**
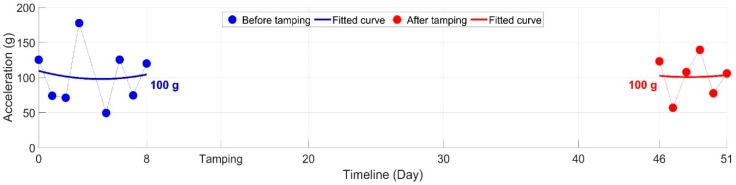
Development of the wheel-rail impacts before and after ballast tamping.

The development of the wheel-rail impacts before and after tamping are presented in Figure 15. In this figure, each point represents the mean value of the impact accelerations based on multiple wheel passages in one monitoring day. It was discussed in a previous study [28] that the fluctuation of the wheel impacts was highly affected by external disturbances such as the weather. Still, it can be seen that the regression values before and after tamping were both around 100 g. 

It can be concluded that such frequently implemented ballast tamping had no improvement in either the ballast condition or the dynamic performance of the monitored crossing. Without figuring out the root causes for the fast crossing degradation, such ineffective ballast tamping should be suspended.

### 4.2. Fastening System Renovation

**Figure 16:**
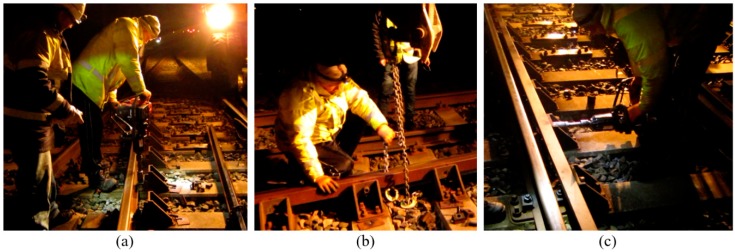
Fastening system renovation. (**a**) Remove the broken bolts. (**b**) Reposition the guard rail. (**c**) Install new bolts.

During the monitoring period, the fastening system was found to be degraded with some broken bolts. Such degradation affected the lateral stability of the track. Therefore, the fastening system, mainly the bolts in the guard rails and the clips, was renovated, as shown in Figure 16.

**Figure 17:**
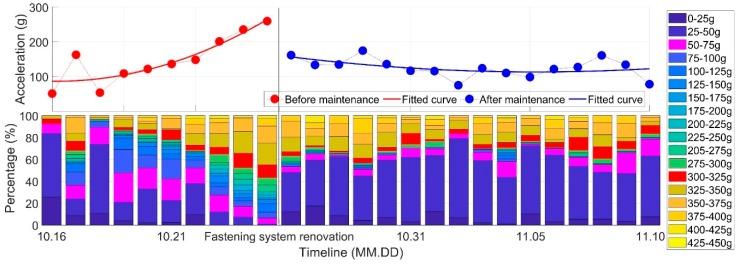
Effect of fastening system renovation on the dynamic performance of the crossing.

The development of the wheel-rail impacts before and after renovation is shown in Figure 17. The upper figure is the development of the mean value, and the lower figure gives the ratio of different impact levels in each monitoring day, corresponding to the value in the upper figure.

It can be seen from Figure 17 that before the renovation, the wheel-rail impact showed a clear increasing trend with the impact values widely distributed from 0 to 450 g. Such a degradation trend indicates that maintenance is urgently required due to the defects of the fastening system. After the renovation, the wheel-rail impacts were dramatically reduced in terms of the mean value and separated into two distribution modes, which is similar to those shown in Figure 11a. Such improvement is due to the enhancement in the track integrity. However, the wheel-rail impacts above 300 g were still a large proportion after maintenance, which means that the sources for such high wheel-rail impacts were not found.

In practice, ballast tamping is currently one of the few options for contractors to maintain the track. However, the unimproved crossing performance clearly indicates the ineffectiveness of tamping. The fastening system renovation was a forced action to repair damaged components. Although the crossing performance was improved, the extremely high wheel-rail impacts were not reduced, thus the sources for the fast crossing degradation were not eliminated. To figure out the root causes for the crossing damage, the track inspection was extended to the bridge in front of the crossing (Figure 10b). The results for the track inspection, as well as the numerical verification using the MBS model, are presented in the next section.

## 5. Damage Sources Investigation

In this section, the track inspection, including the whole turnout and the adjacent bridge, is presented. The inspected degradations will be input into the MBS model to verify the influence on the crossing performance. As a reference, the dynamic responses in the designed condition with no track degradations were also simulated and compared with those in degraded conditions. The verification results, followed by the analysis, are also presented.

### 5.1. Track Inspection

**Figure 18:**
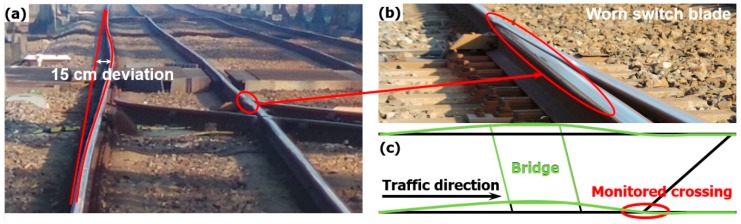
Track deviation in front of the crossing. (**a**) Inspected curve introduced by the bridge. (**b**) Worn switch rail. (**c**) Demonstration of the bridge deviation.

In the field inspection, it was found that the bridge was not well aligned in the track, but deviated around 15 cm, as shown in Figure 18a. Such deviation introduced a curve into the track, which was likely to be out of design since no elevation was set up in the outer rail. It can be imagined that the passing trains could not pass the track along the central line but tended to have wheel flange contact with the outer rail, which eventually leads to the severe wear in the switch blade (Figure 18b).

The accumulated effect of the track deviation was also reflected in the variated track gauge. It was shown in the measurement results that the gauge variations along the whole turnout were up to 3 mm, as presented in Table 1. Considering that the monitored crossing is located quite close to the bridge (Figure 18c), such track misalignment, including the track deviation in the bridge and track gauge variation along the turnout, may affect the wheel-rail interaction in the crossing.

### 5.2. Numerical Verification and Analysis

**Figure 19:**
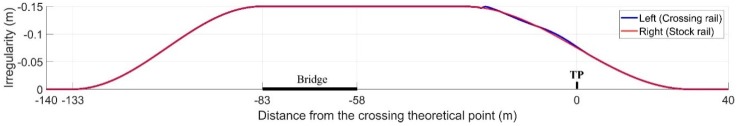
Equivalent lateral irregularities in the track.

In order to verify the effect of the track lateral misalignment on the performance of the crossing, both the bridge-introduced curve and the track gauge variation were input into the MBS vehicle-crossing model (Figure 9). The equivalent track lateral irregularities as the model input are shown in Figure 19.

In the MBS model, the crossing type is the same as the monitored 1:9 crossing with the rail type of UIC54 E1. The vehicle model is consistent with the recorded VIRM train with the wheel profile of S1002. The initial track parameters of Dutch railways [32] applied in the model are given in Table 2.

**Figure 20:**
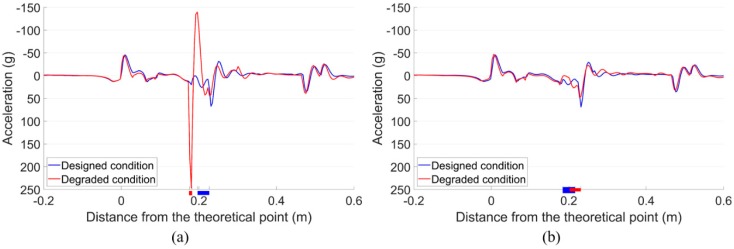
Vertical impact acceleration responses and transition regions. (**a**) Front wheel. (**b**) Rear wheel.

With the track misalignment taken into account, the crossing condition was considered as degraded. The simulation results of both wheels in the bogie, including the wheel impact accelerations and transition regions, were compared with the results in the designed condition [29], as shown in Figure 20.

It can be seen from Figure 20a that with the lateral irregularity taken into account, the impact of the front wheel was dramatically increased to 247 g, which was 4 times higher than the reference value (around 62 g) in the designed condition. While for the rear wheel from the same bogie, the impact was 48 g, which was even lower than the reference value. Despite the slight difference in the absolute values, the simulation results were consistent with the measurement results (Figure 11). Meanwhile, the transition region of the front wheel was 0.176–0.182 m from the TP with a size of only 0.006 m. Compared with the reference level (0.196–0.217 m with a size of 0.031 m, [29]), it was much narrower and closer to the TP, indicating earlier wheel impact and much sharper wheel load transition in the crossing. For the rear wheel, although the transition region was located farther from the TP, the size was almost the same as the reference value.

Such results clearly show that the curve and lateral track misalignment in front of the crossing can lead to unstable wheel-rail contact in the crossing and sometimes result in extremely high impacts. Additionally, the front and rear wheels pass through the crossing quite differently, which indicates that the performance of the rear wheel is not independent, but is affected by the front wheel.

**Figure 21:**
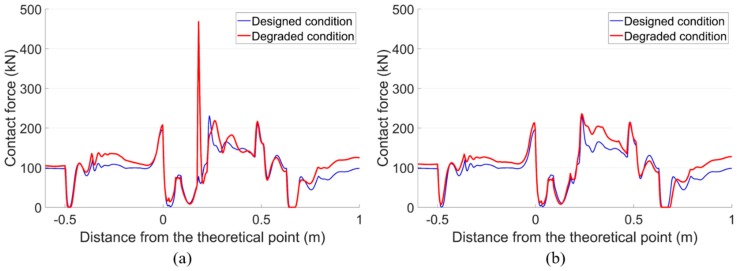
Vertical wheel-rail contact responses of the facing crossing. (**a**) Front wheel. (**b**) Rear wheel.

For the wheel-rail contact forces, the tendency was similar to the acceleration responses, as shown in Figure 21. With the degraded track condition, the maximum contact force of the front wheel in the degraded condition was 468 kN, which was twice as high as that in the designed condition (235 kN). While for the rear wheel, the difference between the degraded condition and the designed condition was limited.

**Figure 22:**
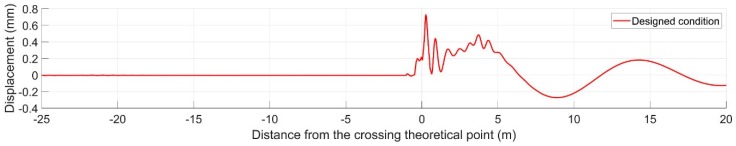
Wheel lateral displacement in the designed condition.

To understand how the track misalignment affects the wheel-rail interaction in the crossing, the relationship between the wheel lateral displacements and wheel-rail contact forces were analyzed. Before that, the wheel lateral displacement in the designed condition is presented in Figure 22. When the train enters the crossing panel, the variated rail geometry will lead to the lateral movement of the wheel. The maximum lateral displacement was around 0.7 mm.

**Figure 23:**
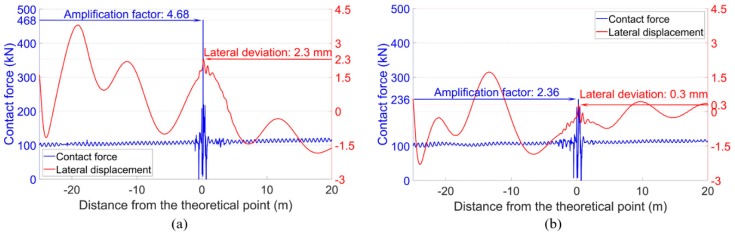
Wheel-rail contact forces and wheel lateral displacements. (**a**) Front wheel. (**b**) Rear wheel.

In the degraded condition with track lateral irregularities, the lateral displacements of the wheels were dramatically changed, as shown in Figure 23. It can be seen that both the front wheel and the rear wheel showed activated hunting oscillation before and after passing through the crossing, but the trajectories were quite different. For the front wheel, the lateral movement was more intense and ran toward the crossing nose rail near the TP. The maximum lateral displacement corresponding to the position with the highest contact force was 2.3 mm, which means that compared with that in the designed condition, the wheel flange was around 1.6 mm closer to the nose rail. Comparatively speaking, such displacement of the rear wheel was only 0.3 mm. Such results indicate that the wheel-rail impact was profoundly affected by the movement of the wheel. When the wheel approaches closer to the crossing nose, the wheel-rail impact is likely to be increased. It can be concluded that the train hunting activated by the lateral track misalignment in front of the crossing is the main cause of the extremely high wheel-rail impacts. 

The train hunting effect also explains the unstable wheel-rail impacts. For the rear wheel, the lateral movement was affected not only by the track misalignment but also by the front wheel from the same bogie. As a result, these two wheels led to quite different wheel trajectories. It can be imagined that in the real-life situation, there are much more factors that may affect the hunting motion of each passing wheelset such as the initial position of the wheel when entering the misaligned track section, the mutual interaction between the adjacent wheelsets, the lateral resistance of the track, and even the weather condition [28], etc. The combined effect of all these factors ultimately resulted in the polarized distribution of the impact acceleration responses (Figure 11a).

### 5.3. Respective Effect of Lateral Curve or Track Gauge Deviation

It can be noticed that in the previous analysis, the input track misalignment consisted of two parts: the lateral curve introduced by the bridge and the track gauge deviation. In order to understand the effect of each part in the wheel-rail interaction, these two parts were further analyzed, and the results are presented below.

**Figure 24:**
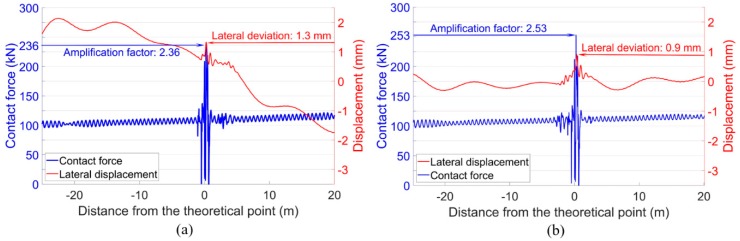
Wheel-rail contact forces and lateral wheel displacements. (**a**) Front wheel. (**b**) Rear wheel.

Considering the bridge-introduced lateral curve, the wheel-rail contact forces and the lateral wheel displacements were calculated, as presented in Figure 24. It can be seen that in the front wheel, the bridge-introduced curve mainly resulted in the lateral shift of the wheel trajectory due to the centripetal force. Such a shift was only 0.5 mm near the crossing nose when compared with the designed condition, and the effect on the wheel impact was limited. For the rear wheel, the combined effect of the curve and the motion of the front wheel resulted in the lateral deviation of 0.9 mm, which was quite close to that in the designed condition and had no significant influence on the wheel-rail impact.

**Figure 25:**
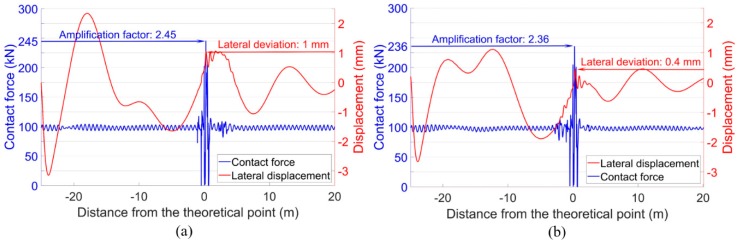
Wheel-rail contact forces and wheel lateral displacements. (**a**) Front wheel. (**b**) Rear wheel.

The effect of the track gauge deviation on the wheel-rail interaction is demonstrated in Figure 25. Different from the effect of the bridge-introduced curve, the deviated track gauge activated the hunting motion of the passing wheels. Still, the resulted lateral wheel displacements were not large enough to amplify the wheel-rail impact. The maximum displacements corresponding to the wheel impacts were 1 mm in the front wheel and 0.4 mm in the rear wheel, respectively.

### 5.4. Summary

Based on the above analysis, it can be concluded that the extremely high wheel-rail impacts in the monitored crossing were caused by the hunting oscillation of the passing trains. Such train hunting was the combined effect of the bridge-introduced curve in front of the crossing and the deviated track gauge along the turnout. When the maximum wheel lateral displacement reaches a certain level (e.g., 2.3 mm), the wheel-rail impact will be dramatically amplified.

It has to be noted that although the curve in front of the crossing did not directly activate train hunting, the activated lateral shift of the passing wheels resulted in the wear in the switch blade (Figure 18b) and contributed to the track gauge deviation. Therefore, such a curve can be considered as the root cause of the fast degradation of the monitored crossing. To improve the performance of the crossing, this curve has to be first eliminated.

In the previous study [28], it was proven that high rail temperature due to the long duration of sunshine would amplify the existing track geometry deviation in turnout and lead to the increase in the wheel-rail impacts. The train hunting activated by the track gauge deviation in this study further confirmed these results.

## 6. Effect of Maintenance-Related Degradation

According to the measurement results, the monitored crossing also suffered from ballast settlement and broken clips. In order to better simulate the real-life situation, these track defects were respectively added to the degraded MBS model developed in Section 5.2. The combined effects were simulated and analyzed, as presented below.

### 6.1. Effect of Ballast Settlement

**Figure 26:**
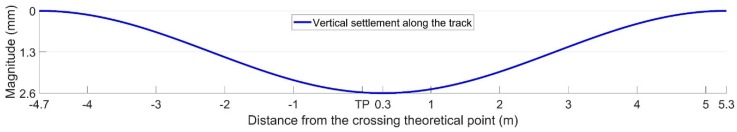
Ballast settlement introduced in the MBS model.

It is shown in Figure 13 that the detected ballast settlement was around 2.6 mm. To simplify the problem, a vertical irregularity was introduced in the MBS model to simulate the ballast settlement, as shown in Figure 26. In this irregularity function, the amplitude was 1.3 mm, and the wavelength was 10 m. The trough of the wave was located 0.3 m from the TP of the crossing, which was consistent with the instrumented accelerometer and the installed displacement target.

**Figure 27:**
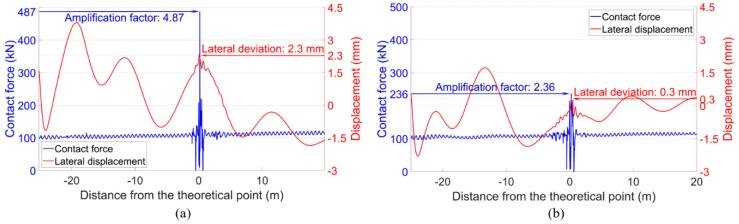
Wheel-rail contact forces and lateral wheel displacements. (**a**) Front wheel. (**b**) Rear wheel.

With the ballast settlement taken into account in the MBS model, the dynamic performance of the crossing was simulated. The representative results are shown in Figure 27. It can be seen that the simulation results were almost the same as those without ballast settlement (Figure 23), despite the slightly increased impact force of the front wheel (from 468 kN to 487 kN). It can be concluded that the existence of ballast settlement had a limited influence on the dynamic performance of the crossing. From another point of view, the ballast settlement was more likely to be the accumulated effect of the high wheel-rail impacts. Such results further explain the ineffectiveness of the frequently performed ballast tamping since ballast settlement is not the main cause of the extremely high wheel-rail impacts.

### 6.2. Influence of Reduced Lateral Support

**Figure 28:**
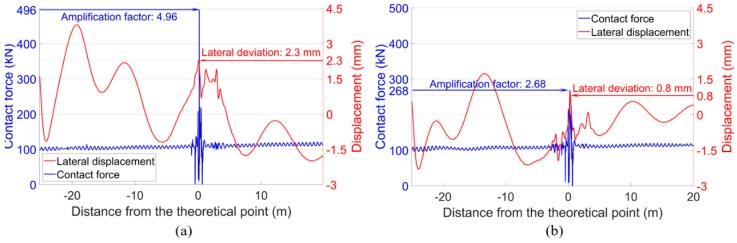
Wheel-rail contact forces and lateral wheel displacements. (**a**) Front wheel. (**b**) Rear wheel. Note: Ballast settlement was not taken into account.

It is shown in Figure 16 that the defects of the fastening system can increase the instability of the wheel-rail impact in the crossing. Combined with the maintenance action and the simulation results in Section 5, it can be inferred that this effect was caused by the reduced lateral track resistance. To verify this inference in the degraded model (Section 5.2), the input lateral stiffness of the clips in the crossing panel was reduced from 280 MN/m (Table 2) to 2.8 N/m, and the corresponded damping was reduced from 580 kN·s/m to 5.8 N·s/m. Based on these inputs, the wheel-rail contact forces and the lateral wheel displacements were calculated, as presented in Figure 28.

It can be seen from Figure 28 that with the reduced lateral stiffness and damping of the clips, the impacts of both the front wheel and the rear wheel were slightly increased (compared with the results in Figure 23). Moreover, the hunting motion of wheels in the crossing panel was more intense. As a result, the lateral deviation of the rear wheel increased from 0.3 mm to 0.8 mm. It can be imagined that with the impacts of the passing trains, the track alignment will continuously be changing due to the reduced structural integrity. The changed track alignment will, in return, act on the wheel-rail interaction and eventually lead to more unstable wheel impacts in the crossing (Figure 17). From this point of view, renovating the defected fastening system is necessary for a monitored crossing. Enough track lateral resistance can help to maintain better crossing performance.

## 7. Conclusions

In this study, the root cause of the fast degradation of a 1:9 crossing in the Dutch railway system was investigated. The effectiveness of some typical track maintenance actions was also assessed and verified. Based on the measurement and simulation results, the following conclusions can be drawn:

- The fast crossing degradation was directly caused by the extremely high wheel-rail impacts, and the root cause for such high impacts was the hunting of the passing trains that were activated by the track lateral misalignment in front of the crossing. When the lateral deviation of the passing wheel exceeds a certain extent (e.g., 2.3 mm), the wheel-rail contact situation will change and the wheel impacts will be dramatically increased. To improve the current situation, such track misalignment needs to be eliminated;
- Ballast settlement is likely to be the accumulated effect of the high wheel-rail impacts. The influence on the crossing performance is somewhat limited. Ballast tamping, especially with only the squeezing machine, cannot improve the dynamic performance of the crossing. In the case of not knowing the sources of damage, it is better to take no action, rather than implement ballast tamping;
- Fastening system renovation helped improved the crossing performance by providing better lateral support in the track but was not targeted to the fundamental problem. Therefore, such damage repair action is useful, but not enough for an improvement in the crossing performance.

This study further verified the effectiveness of the previously proposed condition indicators in the investigation of the damage sources of the crossing. Since the root causes for the fast degradation were the deviated track in front of the crossing, this means that the degradation detection is not only restricted to the crossing itself but can also take the adjacent structures into account.

The activated train hunting reasonably explained the instability of wheel-rail interaction in the crossing, which pointed out a possible direction to maintain the problematic crossings in the Dutch railway network. As part of the Structural Health Monitoring System for railway crossings developed in TU Delft, the findings in this study will help improve the current maintenance philosophy from “failure reactive” to “failure proactive”, and eventually lead to sustainable railway crossings.

## Figures and Tables

**Table 1:** Track gauge measurement results in the critical sections along the turnout.

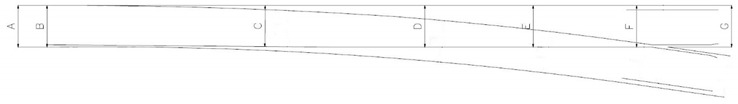							
Location	A	B	C	D	E	F	G
Deviation (mm)	+2	+3	−2	−2	+2	+3	0

**Table 2:** Track parameters.

Track Components		Stiffness, MN/m	Damping, kN·s/m
Rail pad/Clips	Vertical	1300	45
	Lateral	280	580
	Roll	360	390
Ballast	Vertical & lateral	45	32
